# Albendazole–doxycycline combination therapy alleviates MRI- and pathology-evident neuroinflammation and restores IL-33/GFAP balance in mouse neuroangiostrongyliasis

**DOI:** 10.1186/s13071-026-07284-6

**Published:** 2026-02-06

**Authors:** Kai-Yuan Jhan, Eny Sofiyatun, Shao-Chieh Chiu, Chih-Jen Chou, Yi-An Day, Pei-Jui Chiang, Shih-Ming Jung, Wei-June Chen, Po-Ching Cheng, Lian-Chen Wang

**Affiliations:** 1 Department of Parasitology, College of Medicine, Chang Gung University, Taoyuan, 333 Taiwan; 2 Graduate Institute of Biomedical Sciences, College of Medicine, Chang Gung University, Taoyuan, 333 Taiwan; 3 Department of Environmental Health, Polytechnic College of Banjarnegara, Banjarnegara, Central Java 53482 Indonesia; 4 Molecular Imaging Center, Chang Gung Memorial Hospital, Taoyuan, 333 Taiwan; 5 Department of Medical Biotechnology and Laboratory Science, College of Medicine, Chang Gung University, Taoyuan, 333 Taiwan; 6 School of Medicine, College of Medicine, Chang Gung University, Taoyuan, 333 Taiwan; 7 Department of Pathology, Chang Gung Memorial Hospital, 333, Taoyuan, Taiwan; 8 Molecular Infectious Disease Research Center, Chang Gung Memorial Hospital, Taoyuan, 333 Taiwan; 9 Department of Molecular Parasitology and Tropical Diseases, School of Medicine, College of Medicine, Taipei Medical University, Taipei, Taiwan; 10 Graduate Institute of Medical Science, College of Medicine, Taipei Medical University, Taipei, Taiwan; 11 Center for International Tropical Medicine, College of Medicine, Taipei Medical University, Taipei, Taiwan

**Keywords:** Angiostrongyliasis cantonensis, Albendazole, Doxycycline, Co-treatment, Magnetic resonance imaging, GFAP, IL-33

## Abstract

**Background:**

*Angiostrongylus cantonensis* (rat lungworm) infection causes neuroangiostrongyliasis, a parasitic disease characterized by eosinophilic meningitis and meningoencephalitis. Within the central nervous system (CNS), larval migration and degeneration provoke neuroinflammation involving microglia and astrocytes. Albendazole (ABZ) is the mainstay treatment but may exacerbate inflammation through antigen release from dying worms. Doxycycline (DOX), a tetracycline antibiotic with anti-inflammatory and neuroprotective properties, can attenuate glial activation and matrix metalloproteinase activity. As a follow-up to our previous work on ABZ–DOX treatment outcomes, this study evaluated whether ABZ–DOX co-therapy (co) provides antiparasitic and neuroprotective benefits associated with interleukin (IL)-33/glial fibrillary acidic protein (GFAP) regulation in *A. cantonensis*-infected mice.

**Methods:**

A laboratory-maintained Taiwan strain of *A. cantonensis* was used to infect 7–8-week-old C57BL/6 and BALB/c mice (50 third-stage larvae/mouse). For terminal analyses (histopathology, western blotting, and enzyme-linked immunosorbent assay [ELISA]), animals were allocated to eight groups: uninfected control, infected untreated, early ABZ (7–21 days post infection [dpi]), late ABZ (14–21 dpi), early DOX (7–21 dpi), late DOX (14–21 dpi), early ABZ–DOX co-therapy (co; 7–21 dpi), and late co-therapy (co; 14–21 dpi); all were euthanized at 21 dpi. Parasite recovery was performed in an independent cohort following the early-treatment schedule. Magnetic resonance imaging (MRI; 7.0 T) was conducted in a separate longitudinal BALB/c cohort (infected untreated versus early co) scanned up to 28 dpi. Statistical analyses were conducted using *t*-tests.

**Results:**

In an independent cohort treated using the early schedule (7–21 dpi), ABZ-containing regimens reduced worm recovery to near-zero levels in both strains. Histopathology showed eosinophilic meningitis, perivascular inflammation, and hemorrhagic changes in infected brains; these lesions were reduced in treated groups, with the most consistent improvements observed in the early co-therapy group relative to infected untreated controls. In a separate longitudinal MRI cohort (BALB/c; infected untreated versus early co-therapy), T2-weighted images demonstrated reduced hyperintensity and edema-like signal changes after early co-therapy. Western blot analyses indicated infection-associated GFAP upregulation and IL-33 alterations across brain regions, whereas co-therapy shifted these markers toward uninfected levels in a region- and strain-specific manner. Serological ELISA showed increased *A. cantonensis*-specific immunoglobulin (Ig)A/G/M reactivity in infected mice, which was reduced in treated groups.

**Conclusions:**

ABZ–DOX co-therapy was associated with reduced parasite recovery and multilevel improvements across pathology, MRI, and glial markers in mice neuroangiostrongyliasis. These findings support ABZ–DOX co-therapy as a candidate regimen for further investigation in the management of *A. cantonensis*-associated neuroinflammation.

**Graphical Abstract:**

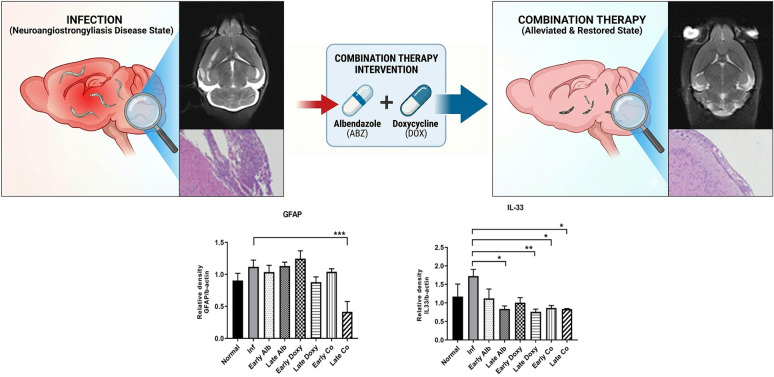

## Background

*Angiostrongylus cantonensis*, commonly referred to as the rat lungworm, is a metastrongyloid nematode responsible for human neuroangiostrongyliasis, an infection that primarily targets the central nervous system (CNS). Since the first human case was documented in Taiwan in the 1940s, this parasite has been increasingly recognized as a major etiological agent of eosinophilic meningitis and meningoencephalitis across tropical and subtropical regions [1–3]. Transmission usually occurs when humans inadvertently ingest the infective third-stage larvae (L3) from contaminated or undercooked intermediate hosts such as *Pomacea canaliculata* or *Achatina fulica* [4, 5]. After ingestion, the larvae penetrate the intestinal wall and migrate through the bloodstream to the brain, where they elicit pronounced inflammatory reactions. Clinically, patients may present with severe headache, fever, paresthesia, or neck stiffness, and cerebrospinal fluid analysis often reveals eosinophilia and elevated protein levels [6].

Within the CNS, *A. cantonensis* induces complex host–parasite interactions that culminate in glial activation and tissue injury. The pathological features include eosinophilic meningitis, perivascular inflammation, hemorrhage, and neuronal loss [7]. These changes result from both the mechanical movement of larvae and the intense immune response triggered by degenerating worms. Activated microglia and astrocytes release multiple cytokines and neuroinflammatory mediators that amplify local tissue damage [8, 9]. In experimental models, strain-specific immune differences have been observed between Th1-dominant C57BL/6 and Th2-dominant BALB/c mice, suggesting that the host’s immunogenetic background shapes disease severity and recovery outcomes [10].

Despite decades of research, the optimal therapeutic regimen for neuroangiostrongyliasis remains unresolved. Albendazole, a benzimidazole derivative that inhibits parasite glucose uptake and tubulin polymerization [11], remains the drug of choice for most helminthic infections. However, the death of worms within the brain can release antigenic debris, exacerbating secondary inflammation and worsening neurological damage [12]. Adjunct corticosteroid therapy may suppress inflammation but fails to remove the parasitic burden. Therefore, novel combination strategies that can concurrently eliminate parasites and suppress neuroinflammatory cascades are of considerable clinical interest [13].

Among antibiotics, doxycycline—a second-generation tetracycline—has drawn attention for its anti-inflammatory and neuroprotective capacities beyond its antimicrobial spectrum [14, 15]. It can modulate microglial activation, inhibit metalloproteinase expression, and attenuate apoptosis in neuronal tissues [16]. Doxycycline’s pharmacokinetic properties, including good CNS penetration and long-established clinical safety, make it an appealing adjunct in inflammatory or infectious neurological disorders such as Alzheimer’s and Parkinson’s diseases [17, 18]. When integrated into antiparasitic regimens, doxycycline may therefore provide synergistic benefits by attenuating host-driven neuroinflammation while albendazole directly eliminates the parasite.

Our previous investigation further supported this hypothesis by demonstrating that albendazole–doxycycline (ABZ + DOX) co-therapy significantly improved cognitive performance and motor coordination in *A. cantonensis*-infected C57BL/6 and BALB/c mice [19]. The same study also revealed strain- and region-specific differences in interleukin (IL)-33 and glial fibrillary acidic protein (GFAP) expression across distinct brain areas under identical treatment conditions, suggesting differential astroglial responses between genetic backgrounds. Building upon these findings, the present work focuses on brain regions showing the most prominent alterations, comparing IL-33 and GFAP expression among treatment groups. To achieve a more comprehensive understanding of the therapeutic impact of ABZ + DOX, we further integrated histopathological evaluation, magnetic resonance imaging (MRI)-based neuroimaging, and peripheral enzyme-linked immunosorbent assay (ELISA) assays to assess whether this combined regimen truly alleviates CNS inflammation and restores neuroimmune balance.

Recent evidence has highlighted that astrocytic IL-33 and glial fibrillary acidic protein (GFAP) play critical roles in coordinating neuroimmune responses following helminth infection [20–22]. IL-33, an alarmin released by astrocytes and endothelial cells, can activate microglia and promote cytokine secretion, whereas GFAP serves as a hallmark of reactive astrocytosis and glial cytoskeletal remodeling [23]. Their interplay may represent a key mechanism underlying CNS inflammation during *A. cantonensis* infection.

Building upon these findings, the present study investigates whether the combined administration of albendazole and doxycycline mitigates neuroinflammation and glial activation in mice neuroangiostrongyliasis. Using both C57BL/6 and BALB/c mice, we assessed the parasitic recovery rate, neuropathological alterations including eosinophilic meningitis and encephalitis, and region-specific brain lesions through MRI. We further examined the expression of IL-33 and GFAP in distinct brain regions and evaluated peripheral immune responses via serum immunoglobulin (Ig)A/G/M ELISA. Collectively, these analyses aim to elucidate host-dependent neuropathological differences and to determine whether doxycycline enhances the therapeutic efficacy of albendazole by reducing glial activation and CNS inflammation during *A. cantonensis* infection.

## Methods

### Establishment and maintenance of the *A. cantonensis* Taiwan strain

A laboratory strain of *A. cantonensis* has been continuously maintained since 1985 to ensure experimental stability and minimize environmental variation. The original isolate was obtained from *Achatina fulica* snails collected in the Neihu District of Taipei, Taiwan. L3 were harvested by artificial digestion of snail tissues, in which minced tissue was incubated in an enzymatic/acidic digestion solution to release larvae, followed by sedimentation and repeated washing to collect intact L3. Approximately 10,000 larvae were then orally inoculated into Sprague–Dawley (SD) rats to complete the parasite life cycle. The first-stage larvae (L1) excreted in the feces of infected rats were subsequently used to infect *Biomphalaria glabrata* snails, allowing continuous propagation of the Taiwan laboratory strain.

L1 were collected from rat feces using a modified Baermann’s technique and introduced into *B. glabrata* snails. At 21 days post infection (dpi), infected snails were homogenized and digested with 0.6% pepsin solution (pH 2.5–3.0) at 37 °C for 45 min. Recovered L3 were counted under a stereomicroscope and used to infect SD rats, maintaining the complete life cycle under controlled laboratory conditions.

### Animals and ethical statement

Specific-pathogen-free SD rats (BioLASCO, Taipei, Taiwan) were employed to sustain the life cycle of *A. cantonensis*, whereas 7–8-week-old BALB/c and C57BL/6 mice (National Center for Biomodels, Taipei, Taiwan) were used for experimental infection. Animals were maintained at 22 ± 2 °C, 55 ± 5% humidity, and a 12 h light/dark cycle with free access to food and water. All animal protocols were reviewed and approved by the Institutional Animal Care and Use Committee (IACUC) of Chang Gung University (IACUC approval no.: CGU107-049).

### Experimental infection

Third-stage larvae (L3) were isolated from infected *B. glabrata* snails as described above. Each mouse was inoculated orally with 50 viable L3 via a soft feeding needle. Mice were monitored daily for behavioral and physical changes until they were sacrificed.

### Drug administration

Both C57BL/6 and BALB/c mice were randomly assigned to eight groups (*n* = 5 per group): uninfected control, infected untreated, early albendazole (ABZ; 7–21 dpi), late ABZ (14–21 dpi), early doxycycline (DOX; 7–21 dpi), late DOX (14–21 dpi), early ABZ–DOX co-therapy (co; 7–21 dpi), and late co-therapy (co; 14–21 dpi). ABZ was administered at 10 mg/kg/day via oral gavage, and DOX at 25 mg/kg/day intraperitoneally. For terminal analyses (histopathology, Western blotting, and ELISA), all animals were euthanized at 21 dpi; uninfected controls were euthanized at the same age. Parasite recovery and MRI were performed in independent cohorts as described below.

### Parasite recovery

Parasite recovery was performed in an independent cohort of infected mice treated using the early-treatment schedule. At 21 dpi, mice were anesthetized with 3% isoflurane and whole brains were excised. Brain tissue was homogenized in saline, and live worms were enumerated under a dissecting microscope. Recovery rates were expressed as the percentage of recovered larvae relative to the original inoculum. Brains used for worm recovery were not used for downstream histology or biochemical assays.

### Collection of serum and brain samples

Blood was obtained via terminal cardiac puncture and allowed to clot at 4 °C overnight. After centrifugation (1300 rpm, 45 min), serum was collected and stored at −80 °C for ELISA. Brains were removed and bisected along the midline: one hemisphere was fixed in 10% neutral-buffered formalin for histology, and the contralateral hemisphere was snap-frozen for Western blot analysis, enabling matched peripheral and CNS readouts from the same animals.

### Histopathology and lesion scoring

Fixed brains were processed and regionally sampled according to the Allen Brain Atlas. Each brain was divided into five regions (anterior cerebrum; lateral ventricles; third ventricle and hippocampus; posterior cerebrum and fourth ventricle; and cerebellum). From each region, five coronal paraffin sections (5 μm) were prepared and stained with hematoxylin and eosin (H&E), yielding a total of 25 sections per mouse for scanning-based analysis. Pathological parameters—including eosinophilic meningitis, perivascular cuffing, encephalitis, larval presence, hemorrhage, and congestion—were semiquantitatively graded as 0 (absent), 1 (≤ median lesion extent), or 2 (> median lesion extent). Histopathological scoring was performed by an investigator blinded to treatment group allocation. For larval findings, scores reflected the number of larval cross-sections observed per section; although repeated counting of the same larva cannot be fully excluded across serial sections, overall parasite burden was quantified independently by worm recovery.

### Western blot analysis

Brain tissues were dissected into the cortex, hippocampus, and cerebellum and homogenized in ice-cold 1% sodium dodecyl sulfate (SDS, PAGE) buffer. Lysates were centrifuged at 13,000 rpm for 30 min at 4 °C, and protein concentrations were determined using the Bio-Rad protein assay kit. Equal amounts of protein (30 µg) were separated by SDS-polyacrylamide gel electrophoresis (PAGE) and electrotransferred to nitrocellulose membranes (GE Healthcare). After blocking with 5% nonfat milk, membranes were incubated overnight at 4 °C with primary antibodies: anti-IL-33 (1:10,000; Abcam, ab187060), anti-GFAP (1:10,000; Abcam, ab7260), and anti-β-actin (1:5,000; Merck/Sigma-Aldrich, A5441). Horseradish peroxidase (HRP)-conjugated anti-rabbit immunoglobulin G (IgG, 1:10,000; Sigma-Aldrich, A0545) served as the secondary antibody. Protein bands were visualized using an enhanced chemiluminescence (ECL) detection system (ChemiDoc XRS + , Bio-Rad).

### Enzyme-linked immunosorbent assay (ELISA)

Serum immunoglobulin responses were quantified by ELISA. Plates were coated overnight at 4 °C with antigens derived from fifth-stage larvae of *A. cantonensis*. After blocking with 1% bovine serum albumin (BSA), diluted serum samples were added and incubated for 1 h at 37 °C. Following phosphate-buffered saline with Tween 20 (PBST) washes, alkaline phosphatase–conjugated goat anti-mouse IgA/G/M secondary antibodies were applied. Color development used *p*-nitrophenyl phosphate (*p*NPP) substrate, and absorbance was read at 405 nm.

### Magnetic resonance imaging (MRI)

MRI was performed in an independent longitudinal cohort of anesthetized BALB/c mice using a preclinical 7-T MRI system (Bruker BioSpin, Germany) equipped with a four-element phased-array mouse brain coil. Mice were positioned in a stereotaxic frame with continuous monitoring of respiration and body temperature. T2-weighted turbo spin-echo (TSE) images were acquired at 0, 7, 14, 21, and 28 dpi (TR 2540 ms; TE 41 ms; inversion time (TI) 0 ms; slice thickness 0.5 mm; field of view 20 × 30 mm^2^; matrix 156 × 320; acquisition time 1 min 54 s). MR images were analyzed in ImageJ (NIH, USA) to assess signal abnormalities (e.g., edema and hemorrhage) and ventricular changes.

### Statistical analyses

Data are expressed as mean ± standard error of the mean (SEM). Differences among the groups were analyzed by Student’s *t*-test. Prism 9 software (GraphPad Software by Dotmatics., San Diego, CA, USA) was used for data integration and graph production.

## Results

### Albendazole–doxycycline co-therapy reduces parasite recovery in both mouse strains

**Fig. 1:**
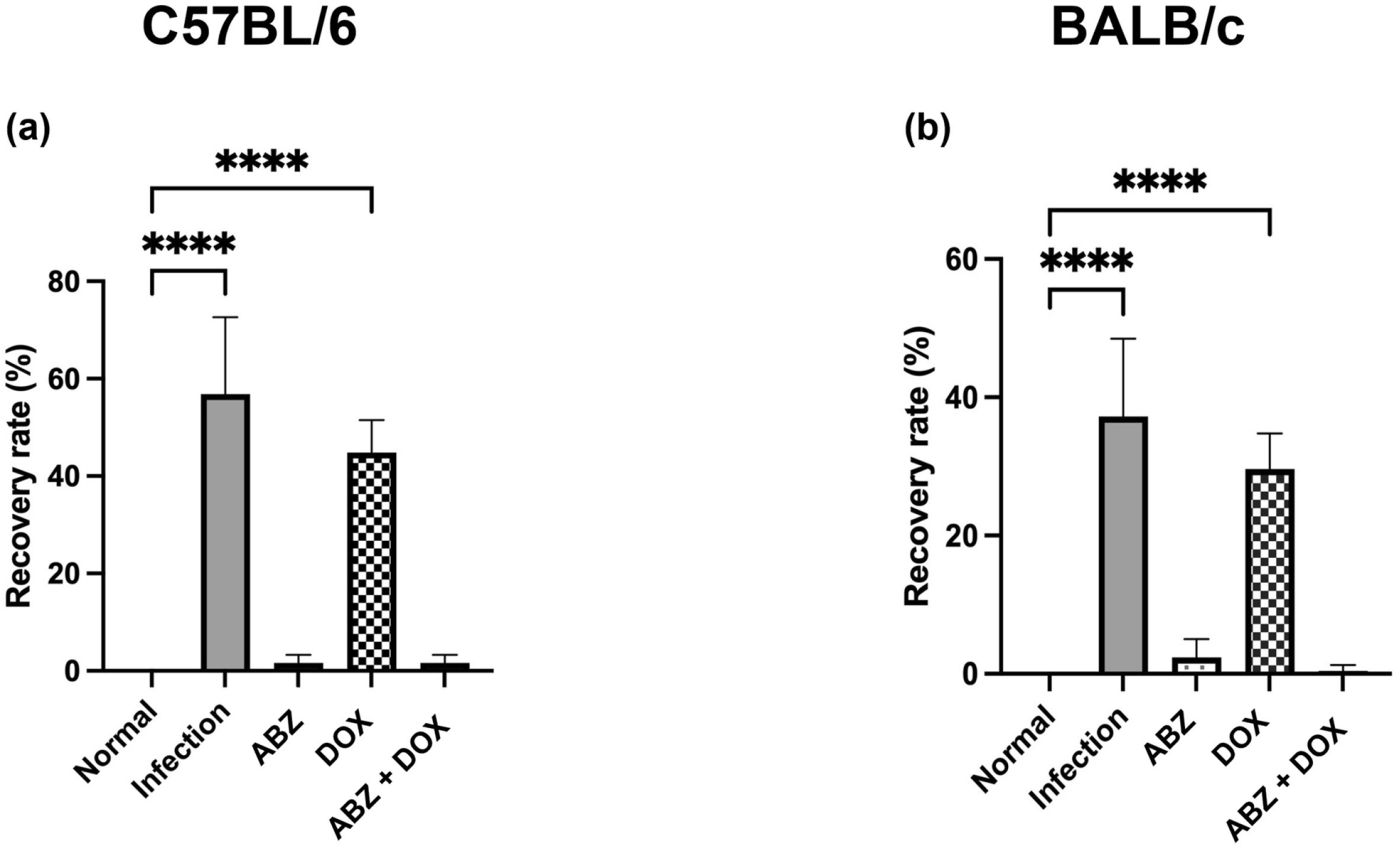
Worm recovery rate in C57BL/6 and BALB/c mice infected with *A. cantonensis* under different therapeutic strategies. **a** C57BL/6 and **b** BALB/c mice were infected with 50 L3 and assessed at 21 days post-infection (dpi). Groups: uninfected control (Normal), infected untreated (Infection), albendazole (Alb), doxycycline (Doxy), and albendazole–doxycycline co-therapy (co). Worm recovery rate is expressed as the percentage of recovered worms relative to the infective dose (*n* = 5 mice/group). Data are mean ± SEM. Statistical comparisons were performed using Student’s *t*-test as indicated on the graph; *P < 0.05, **P < 0.01, ***P < 0.005, ****P < 0.001

Worm recovery was quantified at 21 days post-infection (dpi) in an independent cohort (separate from histology/Western blotting/ELISA) for each strain. In C57BL/6 mice, the infected untreated group showed the highest recovery, whereas albendazole treatment lowered recovery and doxycycline showed a more modest reduction. The albendazole–doxycycline co-therapy group exhibited the lowest recovery, with within-strain statistical differences versus infected untreated indicated in Fig. 1a.

In BALB/c mice, infection likewise yielded measurable recovery, and the infected untreated group again showed the highest recovery. Albendazole, doxycycline, and co-therapy were each associated with reduced recovery, and no worms were detected in the co-therapy group at 21 dpi (Fig. 1b).

### Co-therapy alleviates neuropathological features of meningoencephalitis

**Fig. 2:**
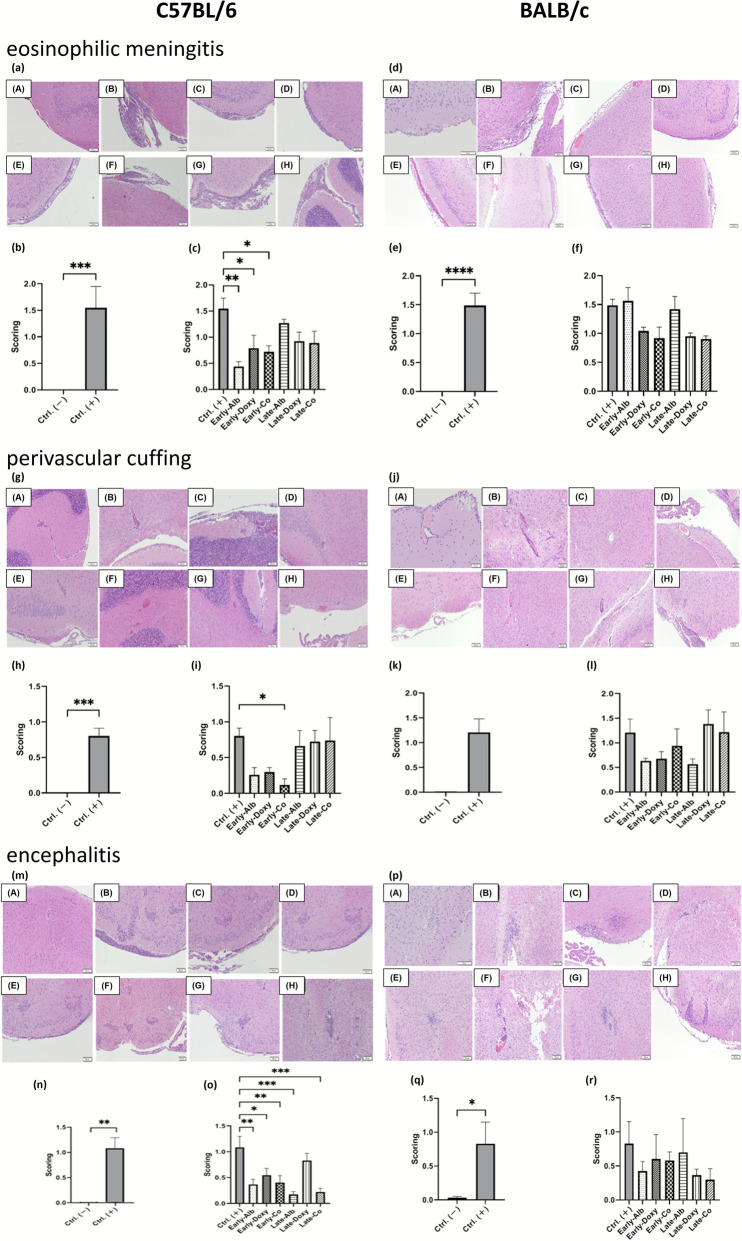
Histopathological evaluation of eosinophilic meningitis, perivascular cuffing, and encephalitis in C57BL/6 and BALB/c mice infected with *A. cantonensis* and treated with different strategies. **a**–**c** Eosinophilic meningitis in C57BL/6 mice: **a** representative H&E-stained sections; **b**,**c** semiquantitative lesion scores. **d**–**f** Eosinophilic meningitis in BALB/c mice: **d** representative sections; **e**,**f** lesion scores. **g**–**i** Perivascular cuffing in C57BL/6 mice: **g** representative sections; **h**,**i** lesion scores. **j**–**l** Perivascular cuffing in BALB/c mice: **j** representative sections; **k**,**l** lesion scores. **m**–**o** Encephalitis in C57BL/6 mice: **m** representative sections; **n**,**o** lesion scores. **p**–**r** Encephalitis in BALB/c mice: **p** representative sections; **q**,**r** lesion scores. In representative panels **a**,**d**,**g**,**j**,**m**,**p**, uppercase letters **A**–**H** denote treatment groups: **A** uninfected; **B** infected untreated; **C** early albendazole (7–21 dpi); **D** early doxycycline (7–21 dpi); **E** early co-therapy (7–21 dpi); **F** late albendazole (14–21 dpi); **G** late doxycycline (14–21 dpi); **H** late co-therapy (14–21 dpi). Each mouse was evaluated across five brain regions, with five coronal sections per region (25 sections/mouse; *n* = 5 mice/group). Scores were assigned as 0 (absent), 1 (≤ median area), or 2 (> median area) as described in “Methods.” Data are mean ± SEM. Statistical comparisons were performed using Student’s *t*-test; *P < 0.05, **P < 0.01, ***P < 0.005, ****P < 0.001

Histopathological examination of H&E-stained brain sections demonstrated neuroinflammatory tissue changes after infection in both strains. Representative sections showed eosinophilic meningitis characterized by meningeal thickening and inflammatory cell infiltration (Fig. 2a, d), with higher meningitis scores in infected untreated mice compared with uninfected controls in both C57BL/6 and BALB/c cohorts (Fig. 2b, e). Across treatment groups, meningitis scores were lower than infected untreated in multiple regimens, including early albendazole and early co-therapy in C57BL/6 mice (Fig. 2c) and both early and late treated BALB/c mice (Fig. 2f).

Perivascular cuffing and encephalitis were evaluated in parallel. Infected untreated mice exhibited increased perivascular inflammation (Fig. 2g, j) and higher perivascular cuffing scores compared with uninfected controls (Fig. 2h, k). Within C57BL/6 mice, early co-therapy was associated with lower scores versus infected untreated (Fig. 2i). Encephalitic lesions, reflected by higher encephalitis scores in infected untreated animals (Fig. 2n, q), were attenuated in select treatment groups, including early and late co-therapy in C57BL/6 mice (Fig. 2o) and multiple treated groups in BALB/c mice (Fig. 2r).

**Fig. 3:**
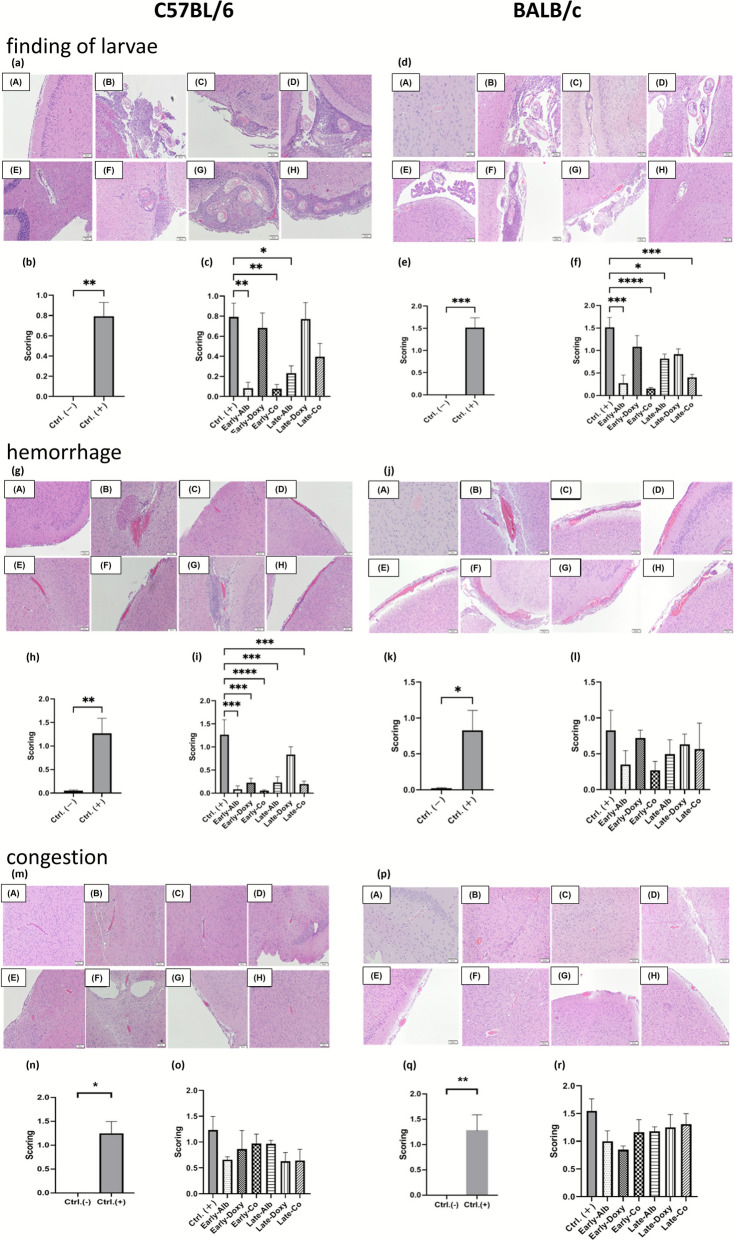
Histopathological evaluation of larval findings, hemorrhage, and congestion in the brains of C57BL/6 and BALB/c mice infected with *A. cantonensis* and treated with different strategies. **a**–**c** Larval findings in C57BL/6 mice: **a** representative H&E-stained sections; **b**,**c** semiquantitative scores. **d**–**f** Larval findings in BALB/c mice: **d** representative sections; **e**,**f** scores. **g**–**i** Hemorrhage in C57BL/6 mice: **g** representative sections; **h**,**i** scores. **j**–**l** Hemorrhage in BALB/c mice: **j** representative sections; **k**,**l** scores. **m**–**o** Congestion in C57BL/6 mice: m representative sections; **n**,**o** scores. **p**–**r** Congestion in BALB/c mice: **p** representative sections; **q**,**r** scores. In representative panels **a**,**d**,**g**,**j**,**m**,**p**, uppercase letters **A**–**H** denote treatment groups: **A** uninfected; **B** infected untreated; **C** early albendazole (7–21 dpi); **D** early doxycycline (7–21 dpi); **E** early co-therapy (7–21 dpi); **F** late albendazole (14–21 dpi); **G** late doxycycline (14–21 dpi); **H** late co-therapy (14–21 dpi). Each mouse was evaluated across five brain regions, with five coronal sections per region (25 sections/mouse; *n* = 5 mice/group). Larval findings were scored based on the number of larval cross-sections per microscopic field as described in Methods. Data are mean ± SEM. Statistical comparisons were performed using Student’s *t*-test; *P < 0.05, **P < 0.01, ***P < 0.005, ****P < 0.001

Additional structural readouts included larval findings, hemorrhage, and congestion. Representative sections illustrated larval cross-sections and migration-associated tissue disruption in infected untreated brains (Fig. 3a, d), along with hemorrhagic foci (Fig. 3g, j) and vascular congestion (Fig. 3m, p). Consistent with these observations, lesion scores were higher in infected untreated mice than in uninfected controls (Fig. 3b, e, h, k, n, q), while multiple treatment arms showed lower scores across the corresponding endpoints (Fig. 3c, f, i, l, o, r).

Across the histopathological endpoints, comparisons were performed within each mouse strain (as displayed in the corresponding subfigures), and the data were not used to infer direct between-strain differences. In several lesion-score endpoints, statistical significance versus infected untreated was observed more consistently in the early co-therapy group than in the late co-therapy group; however, direct early–late comparisons were not performed.

### MRI confirms suppression of neuroinflammatory edema after early co-therapy

**Fig. 4:**
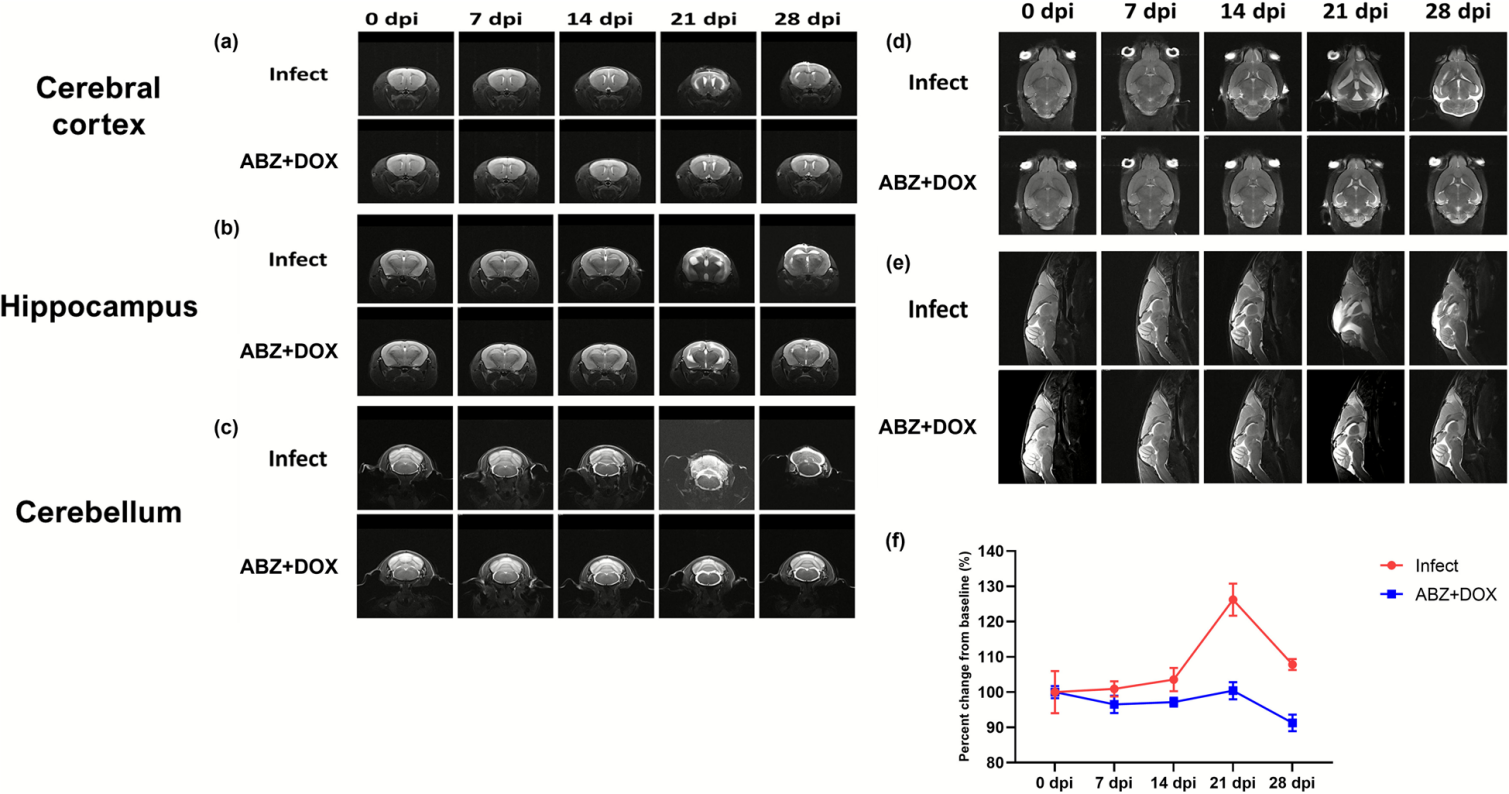
Longitudinal T2-weighted MRI of BALB/c mouse brains following *A. cantonensis* infection and early co-therapy. Serial T2-weighted scans were acquired at 0, 7, 14, 21, and 28 dpi. Panels **a**–**c** show transverse sections at the level of the cerebral cortex, hippocampus, and cerebellum. Panels **d** and **e** show coronal and sagittal views. Panel **f** shows the overall longitudinal quantification across 0–28 dpi, plotted as percent change from baseline (0 dpi) (%). Group labels indicate infected untreated and early co-therapy (ABZ + DOX). MRI parameters: TR 2540 ms; TE 41 ms; inversion time (TI) 0 ms; field of view 20 × 30 mm^2^; matrix 156 × 320; acquisition time 1 min 54 s

MRI was performed in an independent longitudinal cohort of BALB/c mice, with serial scans at 0, 7, 14, 21, and 28 dpi. In infected untreated animals, T2-weighted images revealed a progressive increase in hyperintense signals accompanied by ventricular enlargement, which became most evident at 14–21 dpi in transverse sections at the levels of the cerebral cortex, hippocampus, and cerebellum (Fig. 4a–c), and was further supported in coronal and sagittal views (Fig. 4d, e). Consistently, overall longitudinal quantification expressed as percent change from baseline (0 dpi) (%) showed an increasing trajectory over 0–28 dpi, with the largest elevation occurring around 14–21 dpi (Fig. 4f).

In contrast, mice receiving early albendazole–doxycycline co-therapy exhibited less prominent T2 hyperintensity and a reduced tendency toward ventricular dilation over time across the same transverse levels (Fig. 4a–c), with concordant attenuation in coronal and sagittal planes (Fig. 4d, e) and milder changes by 28 dpi compared with infected untreated mice (Fig. 4a–e). This attenuation was corroborated by the longitudinal quantification, which demonstrated a blunted percent-change-from-baseline trajectory in the co-therapy group across 0–28 dpi (Fig. 4f).

Together, these longitudinal imaging findings support suppression of infection-associated edema by early co-therapy and complement the terminal histopathological readouts acquired at 21 dpi.

### IL-33/GFAP signaling shows region-specific modulation with strain-associated patterns

**Fig. 5:**
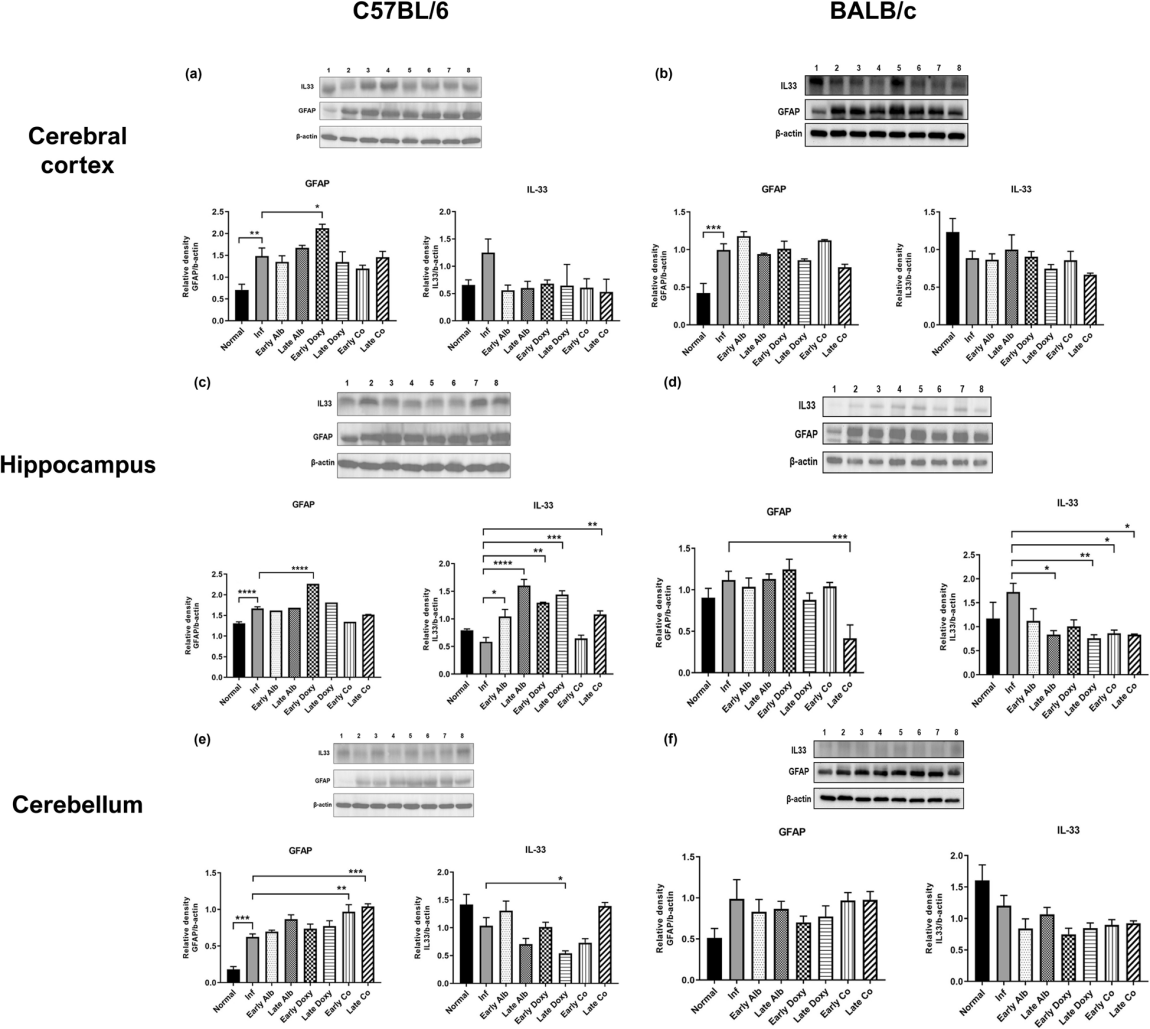
Western blot analysis of GFAP and IL-33 expression across brain regions in C57BL/6 and BALB/c mice infected with *A. cantonensis* and treated with different strategies. Protein expression was assessed in the cerebral cortex (**a**,**b**), hippocampus (**c**,**d**), and cerebellum (**e**,**f**). Lane labels 1–8 correspond to: (1) uninfected; (2) infected untreated; (3) early albendazole (7–21 dpi); (4) late albendazole (14–21 dpi); (5) early doxycycline (7–21 dpi); (6) late doxycycline (14–21 dpi); (7) early co-therapy (7–21 dpi); (8) late co-therapy (14–21 dpi). Densitometric quantification was normalized to β-actin and expressed relative to the uninfected group (*n* = 5 mice/group). Data are mean ± SEM. Statistical comparisons were performed using Student’s *t*-test; *P < 0.05, **P < 0.01, ***P < 0.005, ****P < 0.001

We assessed astrocyte-associated GFAP and IL-33 levels by Western blotting in the cerebral cortex, hippocampus, and cerebellum of C57BL/6 and BALB/c mice across early and late treatment strategies (Fig. 5a–f).

In the cerebral cortex, GFAP was elevated in infected untreated animals compared with uninfected controls in both strains, and treatment groups generally maintained elevated GFAP levels (Fig. 5a, b). In contrast, cortical IL-33 changes were modest and did not show a consistent pattern of significant differences across groups (Fig. 5a, b).

IL-33 displayed clear region-specific patterns, and hippocampal IL-33 responses differed descriptively between strains across treatment groups. In C57BL/6 hippocampus, IL-33 levels in several treatment groups were higher than in infected untreated mice (Fig. 5c), whereas in BALB/c hippocampus, IL-33 was higher in infected untreated mice than in uninfected controls and tended to be lower in treated groups (Fig. 5d). GFAP remained elevated after infection in both strains, with a reduction observed in the BALB/c late co-therapy group (Fig. 5d).

In the cerebellum, GFAP increased after infection in C57BL/6 mice and showed additional treatment-associated variation, while IL-33 exhibited heterogeneous responses, including a lower level in the late doxycycline group (Fig. 5e). In BALB/c cerebellum, IL-33 was lower in infected untreated mice than in uninfected controls and remained relatively low across treatment groups (Fig. 5f).

### Co-therapy is associated with reduced *A. cantonensis*-specific serological reactivity during infection

**Fig. 6:**
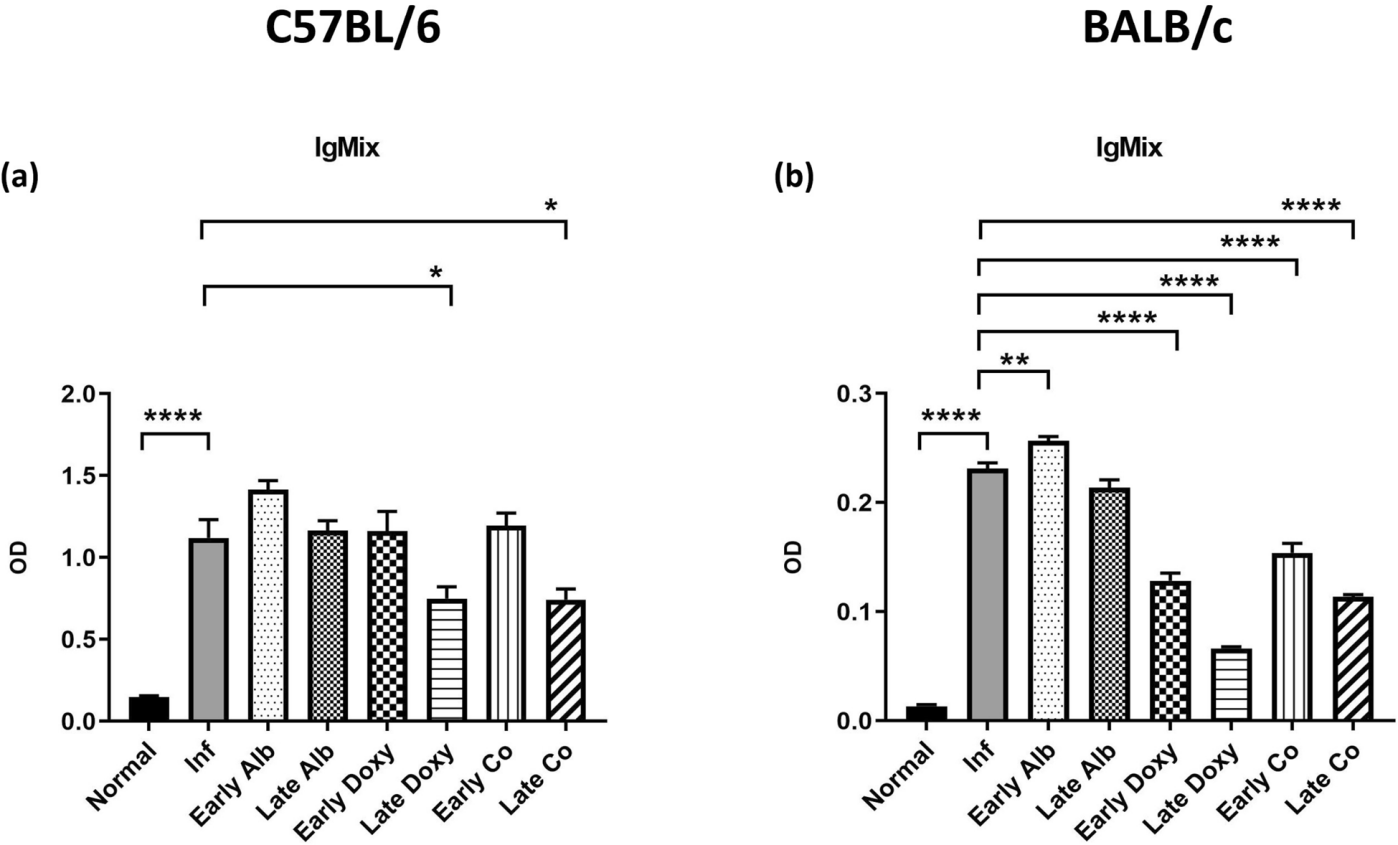
Serological ELISA analysis of *A. cantonensis*-specific immunoglobulin reactivity at 21 dpi. Combined IgA/G/M (IgA/G/M mix) reactivity in serum is shown for C57BL/6 (**a**) and BALB/c (**b**) mice across treatment groups: uninfected; infected untreated; early albendazole (7–21 dpi); late albendazole (14–21 dpi); early doxycycline (7–21 dpi); late doxycycline (14–21 dpi); early co-therapy (7–21 dpi); and late co-therapy (14–21 dpi) (*n* = 5 mice/group). Data are mean ± SEM. Asterisks indicate within-strain comparisons versus uninfected controls (for infected untreated) and versus infected untreated controls (for treatment groups), as indicated on the graph. Statistical comparisons were performed using Student’s *t*-test; *P < 0.05, **P < 0.01, ***P < 0.005, ****P < 0.001

Serum IgA/G/M mix reactivity against *A. cantonensis* was quantified by ELISA at 21 dpi (Fig. 6). In both C57BL/6 and BALB/c mice, infection increased IgA/G/M reactivity relative to uninfected controls (Fig. 6a,b, ****P < 0.001).

Within C57BL/6 mice, late doxycycline and late co-therapy showed lower ELISA signals than infected untreated mice (Fig. 6a, *P < 0.05). Within BALB/c mice, most treatment regimens showed reduced ELISA signals relative to infected untreated animals (Fig. 6b, ****P < 0.001 as indicated), whereas early albendazole showed a higher signal (Fig. 6b, **P < 0.01).

Together, these serological results provide an additional systemic readout alongside parasite recovery, MRI, and neuropathology.

## Discussion

This study used complementary readouts (worm recovery, histopathology, MRI, Western blotting, and serology) to evaluate albendazole (ABZ) and doxycycline (DOX), alone or in combination, in experimental neuroangiostrongyliasis caused by *A. cantonensis*. Consistent with our previous report [19], the current work extends the evidence base by incorporating longitudinal MRI and by examining the IL-33/GFAP markers of astroglial responses across multiple brain regions, providing an integrated view of parasite burden, tissue-level pathology, and host immune-associated changes.

Worm recovery results indicate that ABZ-containing regimens reduce parasite recovery to near-zero levels, while DOX alone shows limited direct anthelmintic activity (Fig. 1). ABZ is known to suppress nematode survival by disrupting microtubule formation and glucose uptake [11, 24]. However, parasite killing can be accompanied by antigenic release that may intensify inflammatory responses [25, 26], which is particularly relevant when interpreting downstream neuropathology. In contrast, DOX is not primarily an anthelmintic but has been reported to mitigate secondary inflammatory consequences through inhibition of microglial activation, suppression of matrix metalloproteinases, and modulation of interferon-related pathways [14, 27]. Together, these complementary actions provide a plausible rationale for why ABZ–DOX co-therapy (co) can reduce parasite recovery while concurrently attenuating host-driven pathology, aligning with prior behavioral evidence that timely combination therapy helps prevent persistent neuroinflammation and cognitive decline in this model [19]. Because each strain was analyzed against its own controls and no direct between-strain statistical testing was performed, observed differences between C57BL/6 and BALB/c should be interpreted descriptively, and the study was not powered for definitive between-strain comparisons.

Histopathological scoring demonstrated that infection is associated with meningeal and perivascular inflammation, encephalitic changes, and vascular-related findings, and that treated groups show lower scores in multiple within-strain comparisons (Figs. 2–3). Across the histopathological endpoints, early co-therapy tended to show more consistent statistical significance versus infected untreated controls than late co-therapy in several comparisons; however, these observations reflect each regimen’s comparisons with infected untreated controls rather than a direct early-versus-late statistical test. In a separate longitudinal MRI cohort (BALB/c; infected untreated versus early co-therapy), T2-weighted imaging provided an in vivo view of time-dependent signal changes that were less apparent after early co-therapy (Fig. 4). MRI was performed only in BALB/c mice as a longitudinal comparison between infected untreated and early co-therapy, and imaging assessments were primarily qualitative, complemented by an overall longitudinal quantification expressed as percent change from baseline (Fig. 4f), but lacked predefined regional ROI-based metrics (e.g., signal intensity mapping and ventricular volumetry). Future studies would benefit from predefined quantitative metrics (e.g., regional signal intensity and ventricular measures) and from extending MRI to monotherapy groups and C57BL/6 mice to strengthen cross-endpoint comparability. In addition, histological larval scoring is based on larval cross-sections and may include repeated counts of the same larva across serial sections; therefore, overall parasite burden was quantified independently by whole-brain worm recovery.

At the molecular level, infection was associated with increased GFAP and altered IL-33, and treatment shifted these markers toward uninfected levels in several within-strain comparisons (Fig. 5). IL-33 can function as an alarmin in CNS injury, but its directionality may depend on cellular sources, timing, and the balance between release, consumption, and tissue dysfunction; thus, the infection-associated IL-33 patterns observed here may reflect stage- and region-specific regulation rather than a uniform injury response. Region-resolved analyses further support that cortical and hippocampal readouts are informative in this model, given their relevance to neuroinflammation-associated cognitive impairment [10, 25]. These findings are consistent with prior reports that cytokine-linked glial regulation can shape tissue outcomes and recovery trajectories [28, 29].

Although we emphasize IL-33/GFAP as a mechanistically informative marker pair for astroglial responses, DOX is known to exert broader anti-inflammatory actions, including inhibition of matrix metalloproteinases, modulation of microglial activation, and interferon-related pathways [14, 27]. Accordingly, IL-33/GFAP changes in our dataset should be viewed as part of a multipathway response rather than the sole mediator of DOX-associated neuroprotection. Linking molecular and imaging scales, reactive astrocytes can influence neurovascular integrity and water/ion homeostasis at the blood–brain barrier, providing a plausible route by which reduced GFAP-associated astrogliosis could coincide with attenuated edema-like MRI signals, while recognizing that direct BBB-focused assays will be important for future mechanistic validation.

Peripheral immune readouts showed that infection was associated with elevated serological antibody reactivity, consistent with systemic immune activation during helminth infection [30]. In our assay, the serological measurement reflects combined IgA/G/M reactivity rather than individual immunoglobulin concentrations, and treatment-associated changes should therefore be interpreted within that analytical scope. Nonetheless, the parallel assessment of central (histology/MRI/WB) and peripheral (serology) measures provides a framework for examining how parasite reduction and immunomodulation may align over the course of disease and treatment in experimental *A. cantonensis* infection.

Taken together, the present findings support the utility of ABZ–DOX co-therapy as a dual-action strategy that targets both the parasitic source of pathology and host-associated neuroinflammatory processes in experimental neuroangiostrongyliasis. The integrated readouts suggest that earlier intervention can be associated with clearer improvements across multiple endpoints, while the study’s design and analytical constraints (e.g., qualitative MRI, limited MRI group coverage, and histological cross-section-based larval scoring) delineate specific priorities for future work. These results provide a clinically relevant rationale for continued evaluation of ABZ–DOX co-therapy for managing human neuroangiostrongyliasis, with further studies needed to refine imaging quantification, broaden cohort coverage, and establish causal links across molecular, vascular, and structural outcomes.

## Conclusions

Combined albendazole–doxycycline co-therapy effectively eliminates *A. cantonensis*, reduces neuroinflammation, restores IL-33/GFAP balance, and protects neural tissue. This dual approach offers synergistic antiparasitic and neuroprotective benefits for treating neuroangiostrongyliasis.

## Data Availability

The data supporting the conclusions of this article are included within the article.

## Abbreviations

IL-33: Interleukin-33
GFAP: Glial fibrillary acidic protein
CNS: Central nervous system
ABZ: Albendazole
DOX: Doxycycline
co: Albendazole–doxycycline co-therapy (ABZ + DOX)
H&E staining: Hematoxylin and eosin staining
ELISA: Enzyme-linked immunosorbent assay
MRI: Magnetic resonance imaging
IgA/G/M: Immunoglobulin A/G/M
L1: First-stage larvae
L3: Third-stage larvae
